# Design, Synthesis and Biological Evaluation of Jahanyne Analogs as Cell Cycle Arrest Inducers

**DOI:** 10.3390/md18030176

**Published:** 2020-03-23

**Authors:** Baijun Ye, Jianmiao Gong, Qiuying Li, Shiqi Bao, Xuemei Zhang, Jing Chen, Qing Meng, Bolin Chen, Peng Jiang, Liang Wang, Yue Chen

**Affiliations:** 1 The State Key Laboratory of Medicinal Chemical Biology, College of Pharmacy and Tianjin Key Laboratory of Molecular Drug Research, Nankai University, Tianjin 300350, China; yebaijunts@126.com (B.Y.); mengqing19920220@163.com (Q.M.); 2120150647@mail.nankai.edu.cn (B.C.); jiang1921372889@126.com (P.J.); 2 Accendatech Co., Ltd., Tianjin 300384, China; jianmiao.gong@accendatech.com (J.G.); liqiuying@mail.nankai.edu.cn (Q.L.); shiqi.bao@accendatech.com (S.B.); xuemei.zhang@accendatech.com (X.Z.); chenjing@accendatech.com (J.C.); 3 Collaborative Innovation Center of Chemical Science and Engineering, Tianjin 300350, China

**Keywords:** jahanyne, α-fluoromethyl ketone, lipopeptide, G0/G1 phase arrest

## Abstract

Jahanyne, a lipopeptide with a unique terminal alkynyl and OEP (2-(1-oxo-ethyl)-pyrrolidine) moiety, exhibits anticancer activity. We synthesized jahanyne and analogs modified at the OEP moiety, employing an α-fluoromethyl ketone (FMK) strategy. Preliminary bioassays indicated that compound **1b** (FMK–jahanyne) exhibited decreased activities to varying degrees against most of the cancer cells tested, whereas the introduction of a fluorine atom to the α-position of a hydroxyl group (**2b**) enhanced activities against all lung cancer cells. Moreover, jahanyne and **2b** could induce G0/G1 cell cycle arrest in a concentration-dependent manner.

## 1. Introduction

**Figure 1:**
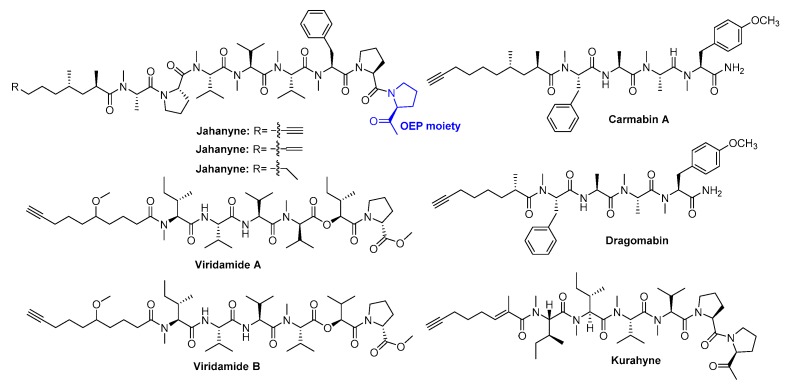
Representative heavily *N*-methylated acyclic lipopeptide with an aliphatic side chain.

In the last few decades, marine cyanobacteria have been identified as rich prolific producers of natural products, bearing a different structure to plant-derived or synthetic bioactive molecules [1,2,3,4]. Moreover, these products exhibit diverse biological activities, ranging from anticancer to antibiotic activity [5,6,7,8]. The discovery of dolastatin-10 [9], cryptophycin-52 [10], and largazole [11] from marine cyanobacteria, and their clinical analogs for cancer therapy, highlights the powerful potential of marine cyanobacteria as a pool for drug discovery [12]. An intriguing lipopeptide-type class from marine cyanobacteria, including dragonamide [13], kurahyne [14], viridamides [15], carmabin A [16], almiramides [17], and jahanyne (Figure 1) [18], is heavily *N*-methylated. Besides this, they share an uncommon terminal alkynyl fatty acid motif. Among these, jahanyne and kurahyne possess a unique OEP (2-(1-oxo-ethyl)-pyrrolidine) moiety. Most of them exhibit a range of antimicrobial, antifungal, and anticancer activities.

Jahanyne was initially isolated by Suenaga et al. as a secondary metabolite from cyanobacteria in 2015 [18]. The data from the initial MTT assays revealed that jahanyne inhibited the growth of both HL60 cells and HeLa cells with an IC_50_ (half-inhibitory concentration) value at the micromolar level and could induce apoptosis in HeLa cells. The bolaamphiphile-mimicking structure of jahanyne and its potent anticancer activity have attracted attention not only from the synthetic community but also from the biological community [19,20,21,22]. Chandrasekhar et al. first completed the total synthesis of desmethyl jahanyne [19]. Preliminary MTT biological assays of desmethyl jahanyne and synthetic intermediates indicated that the *N*-methyl group and terminal alkynyl fatty acid motifs were essential for maintaining jahanyne’s inhibitory activities against cancer cells. The authors further found that, based on a cellular thermal shift assay, several jahanyne intermediates with fatty acid moieties possibly bound to the P2 binding groove on BCL-2 (B cell lymphoma 2), an antiapoptotic protein. Recently, Brimble et al. made a breakthrough in designing the first synthetic route to jahanyne using a modified Fmoc solid-phase synthetic strategy, which could effectively couple multiple sterically-hindered *N*-methylated amino acids without epimerization [20]. Our previous study also provided a convergent and efficient synthetic route supplying us with multi-milligram jahanyne for further biological assays [21]. In the meantime, Suenaga et al. isolated two new jahanyne analogs—jahanane and jahanene [22]—and they achieved their total syntheses. Surprisingly, the MTT assays indicated that the growth-inhibitory activity of jahanyne was only one-tenth of the previously reported activity. They also discovered that a small degree of unsaturation of the terminus of the fatty acid moiety increased growth-inhibitory activity, which might be attributed to the interaction between the P2 binding groove on BCL-2 and the aliphatic chain of the jahanyne family.

**Scheme 1:**
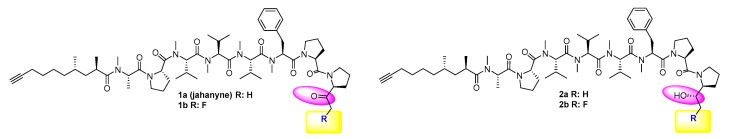
Analogs of jahanyne modified at the OEP moiety.

The introduction of fluorine into amino acids benefits medicinal chemistry in several ways, such as increasing biological profile, interfering with metabolism, and modulating conformation evidenced by pharmaceutical drugs [23,24,25]. Further, α-fluoromethyl ketone (FMK) has been successfully developed as an electrophilic warhead with minimal off-target effects in the development of covalent inhibitors [26,27]. To perform a preliminary evaluation of the structure and activity relationship of OEP moiety and gain a better anticancer lead compound based on jahanyne, we synthesized jahanyne and its analogs (Scheme 1). Herein, we reported our work on synthesis and preliminary biological evaluation of new fluoro analogs of jahanyne.

## 2. Results

### 2.1. Chemistry

**Scheme 2:**
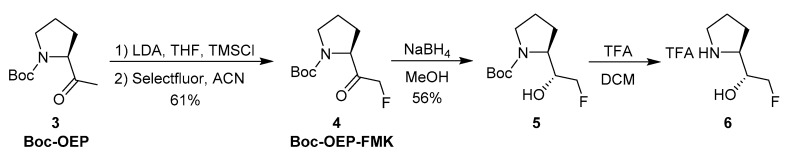
Synthesis of **4**, **5**, and **6**.

Based on the synthetic route we previously developed [21], the modified OEP moiety could be introduced at a late stage. The FMK warhead was constructed, employing commercially available compound Boc–OEP (**3**) as the starting material. The α-deprotonation of **3** with LDA (lithium diisopropylamide) followed by the slow addition of Selectfluor provided us with the Boc–OEP–FMK (**4**) in moderate yield. Then, we reduced the keto group of **4** using NaBH_4_ to provide **5** in 56% yield (Felkin model). Deprotection of Boc with TFA (trifluoroacetic acid) provided us with **6**, which was subjected to a coupling reaction with the acid obtained from the hydrolysis of methyl ester of **7** (Scheme 2).

**Scheme 3:**
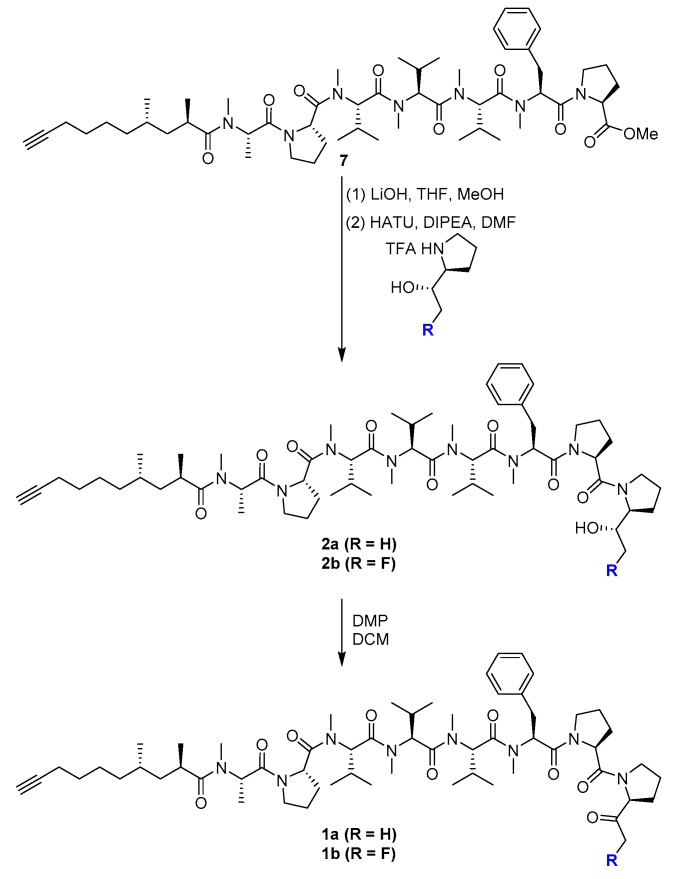
Synthesis of **2a**, **2b**, **1b**, and jahanyne.

Under HATU/DIPEA (1-(Bis(dimethylamino)methylene)-1H-1,2,3-triazolo(4,5-b)pyridinium 3-oxid hexafluorophosphate/*N*,*N*-Diisopropylethylamine) conditions, compounds **2a** and **2b** (for the spectrums of all the synthesized compounds, please see the Supplementary Materials) were obtained in 62% and 65% yields, respectively. Late-stage oxidation of **2a** and **2b** using Dess–Martin periodinane (DMP) followed by instant purification of the crude reaction mixture by column chromatography produced jahanyne (**1a**) and compound **1b** (FMK-jahanyne, Scheme 3).

### 2.2. Biological Activity against Cancer Cells

The in vitro anticancer activities of the four compounds in diverse cancer cells were examined by a Cell Counting Kit-8 (CCK-8) assay.

As shown in Table 1, all the compounds displayed obvious inhibition against cancer cells with moderate to high potencies (IC_50_ = 7.64 − 29.68 μM), whereas compound **2b** presented the most promising activity against H820 cells. A previous study has shown that the natural and synthetic jahanyne exhibit suppression against HL60 cells with IC_50_ values of 4.6 μM and 8.3 μM, respectively [18]. Similarly, the IC_50_ value of the jahanyne (**1a**) synthesized by us against HL60 cells was 13.98 μM. When we applied the FMK strategy using a fluorine atom as a replacement for the hydrogen atom of OEP moiety (**1b**), the inhibitory activity was not improved compared with that of **1a**, except in A549 cells. Compounds **2a** and **2b**, containing a hydroxyl group, showed decreased activities against HL60 cells, and compound **2b** showed a two-fold decrease compared to **1a**. Among H1688 cells, H1299 cells, and H820 cells, compounds **2a** and **2b** showed more potent activities than compounds **1a** and **1b**. Unfortunately, these compounds exhibited no significantly improved activities against A549 cells with IC_50_ values ranging from 13.83 μM to 16.65 μM. It was worth noting that α-fluoromethyl of ketone groups caused decreased activities in varying degrees against H1688 cells, H1299 cells, and H820 cells, whereas the introduction of a fluorine atom to the α-position of the hydroxyl group enhanced activities against all lung cancer cells.

### 2.3. Compound **1a** Induced G0/G1 Phase Arrest

**Figure 2:**
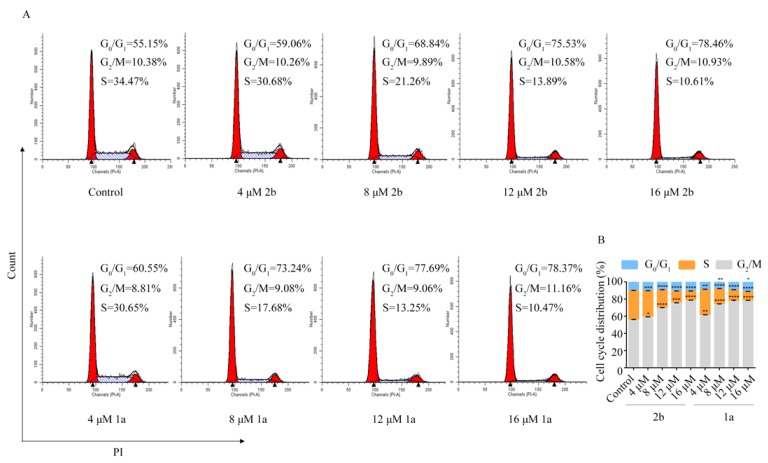
Compounds **2b** and **1a** induced G0/G1 arrest in H820 cells. (**A**) Representative images of cell cycle distribution after treatment of compounds **2b** and **1a** at indicated concentrations. (**B**) Statistical results of cell cycle distribution after treatment of compounds **2b** and **1a**.

To investigate whether the cell growth inhibitory effects of compounds **1a** and **2b** are caused by cell cycle progression, H820 cells were treated with compounds **1a** and **2b** at different concentrations (4, 8, 12, and 16 µM). The cell cycle was analyzed by flow cytometry after staining the DNA with propidium iodide (PI). As shown in Figure 2, after the treatment of compounds **1a** and **2b** for 48 h, the population of cells in the G0/G1 phase increased (78.37% and 78.46%, respectively) compared to the control (55.15%). Inversely, the S phase cell population dramatically decreased. Therefore, these results indicated that compounds **1a** and **2b** could induce G0/G1 phase arrest in a concentration-dependent manner.

### 2.4. Cell Apoptosis Effects of Compound **2b** and **1a**

**Figure 3:**
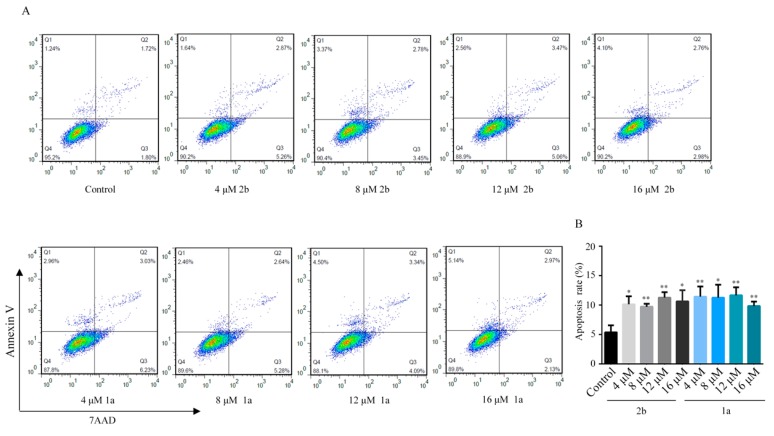
Compounds **2b** and **1a** induced cell apoptosis in H820 cells. (**A**) Representative images of cell apoptosis after treatment of compounds **2b** and **1a** at indicated concentrations. (**B**) Statistical results of cell apoptosis after treatment of compounds **2b** and **1a**.

To evaluate the cell apoptosis effects of compounds **1a** and **2b**, H820 cells were treated with compounds **1a** and **2b** at different concentrations (4, 8, 12, and 16 µM). The cell apoptosis was analyzed by flow cytometry after staining the DNA with 7-aminoactinomycin D (7AAD)/Annexin-V. As shown in Figure 3, compared with the control group, the average rate of apoptosis in H820 cells did not change, but there was a statistical difference. These results suggested that the cell growth inhibitory effects of compounds **1a** and **2b** were not via the induction of apoptosis.

## 3. Materials and Methods

### 3.1. General Information

Reagents and starting materials, purchased from commercial suppliers, were used directly unless otherwise noted. Trifluoroacetic acid (TFA), lithium diisopropylamide (LDA), ethyl acetate (EtOAc), *N*,*N*-dimethylformamide (DMF), *N*,*N*-Diisopropylethylamine (DIPEA), dichloromethane (DCM), Dess-Martin periodinane (DMP), and 1-(Bis(dimethylamino)methylene)-1H-1,2,3-triazolo(4,5-b)pyridinium 3-oxid hexafluorophosphate (HATU).

All reactions were carried out with dry solvents under anhydrous conditions under an argon atmosphere unless otherwise mentioned. Tetrahydrofuran (THF) was distilled from sodium–benzophenone ketyl before using. Reactions were checked by thin-layer chromatography (TLC) on silica gel plates and visualized by UV light and basic aqueous potassium permanganate or aqueous phosphomolybdic acid. Column chromatography was carried out using 200–300 mesh silica gel. Chromatography solvents were purchased from Tianjin Chemical Reagent (Tianjin, China).

Infrared spectra were obtained with a Bruker Tensor 27 instrument (Ettlingen, Germany). Only the strongest and most structurally important absorptions were reported. Optical rotations were measured with an Insmark IP 120 digital polarimeter (Insmark, Shanghai, China). High-resolution mass spectra (HRMS) were obtained on an IonSpec FT-ICR mass spectrometer by Varian 7.0T FTMS (Kuala Lumpur, Malaysia). Further, NMR spectra were recorded in CD_3_OD (δ_H_ = 3.31 ppm, δ_C_ = 49.00 ppm) and CDCl_3_ (δ_H_ = 7.26 ppm, δ_C_ = 77.16 ppm) on a Bruker AV 400, unless otherwise noted. Data for NMR were reported as follows: chemical shift, multiplicity (s = singlet, d = doublet, t = triplet, q = quartet, p = quintet, br = broad, m = multiplet), coupling constants, and integration.

### 3.2. Chemistry

Compound **7** was obtained following the procedure reported previously.

*Tert-butyl (S)-2-(2-fluoroacetyl)pyrrolidine-1-carboxylate* (**4**) 

To the mixture of compound **3** (1.00 g, 4.69 mmol) in THF (15 mL) was added LDA (2.00 M, 2.80 mL, 5.60 mmol) dropwise at −78 °C under argon, and the mixture was stirred for 20 min at −40 °C. A solution of TMSCl (chlorotrimethylsilane) (640 mg, 5.89 mmol) in THF (50 mL) was slowly added dropwise. After stirring for 2 h at −40 °C, the mixture was warmed to −20 °C and stirred for 4 h. The reaction was quenched with saturated aqueous NaHCO_3_ (10 mL) and diluted with EtOAc (30 mL), and the organic layer was separated, washed with brine (3 × 10 mL), dried over Na_2_SO_4_, and filtered. The filtrate was concentrated to afford a silyl enol ether and used directly for the next step. To the solution of the silyl enol ether in CH_3_CN (20 mL) was added Selectfluor (1.66 g, 4.69 mmol). After stirring for 2 h at room temperature, the reaction was quenched with saturated aqueous NaHCO_3_ (10 mL) and diluted with EtOAc (30 mL), and the organic layer was separated, washed with brine (3 × 10 mL), dried over Na_2_SO_4_, and filtered. The filtrate was concentrated, and the residue was purified by silica gel column chromatography (20/1 to 15/1 petroleum ether/EtOAc) to produce compound **4** (662 mg, 61%) as an off-white solid: [α]^17^_D_ −72.4 (*c* 0.1, CHCl_3_); IR (KBr) ν_max_ 2995, 2973, 1682, 1408, 1008 cm^−1^; ^1^H NMR (400 MHz, CDCl_3_) mixture of rotamers δ 5.08, 5.01 (d, *J* = 47.4 Hz, 2H), 4.54–4.43 (m, 1H), 3.54–3.34 (m, 2H), 2.28–2.08 (m, 1H), 1.96–1.82 (m, 3H), 1.43, 1.39 (m, 9H); ^13^C NMR (100 MHz, CDCl_3_) mixture of rotamers δ 205.2 (d, *J* = 16.8 Hz), 205.1 (d, *J* = 17.1 Hz), 154.7, 153.6, 85.4 (d, *J* = 182.1 Hz), 84.9 (d, *J* = 183.2 Hz), 80.6, 80.3, 62.1, 61.6, 46.9, 46.7, 29.8, 28.6, 28.5, 28.4, 24.6, 23.7; ^19^F NMR (400 MHz, CDCl_3_) mixture of rotamers δ −232.3, −232.8 (t, *J* = 49.4 Hz); HRMS (ESI) calculated for [M + Na]^+^ C_11_H_18_FNNaO_3_^+^ was 254.1163 and it was found to be 254.1166.

*Tert-butyl (S)-2-((R)-2-fluoro-1-hydroxyethyl)pyrrolidine-1-carboxylate* (**5**)

To the solution of compound **4** (600 mg, 2.59 mmol) in MeOH (10 mL) was added NaBH_4_ (109 mg, 2.85 mmol) batchwise at 0 °C under argon. The mixture was stirred for 2 h at room temperature and then quenched with water (1 mL) and diluted with EtOAc (30 mL). The organic layer was separated, washed with brine (3 × 5 mL), dried over Na_2_SO_4_, and filtered. The filtrate was concentrated, and the residue was purified by silica gel column chromatography (50/1 to 40/1 petroleum ether/EtOAc) to get compound **5** (339 mg, 56%) as a white solid and isomer tert-butyl (*S*)-2-((*S*)-2-fluoro-1-hydroxyethyl)pyrrolidine-1-carboxylate (**5a**) (109 mg, 18%) as a white solid. Compound **5**: [α]^17^_D_ –77.3 (*c* 0.1, CHCl_3_); IR (KBr) ν_max_ 3433, 2995, 2973, 1682, 1407, 1170 cm^−1^; ^1^H NMR (400 MHz, CDCl_3_) δ 4.49 (ddd, *J* = 25.4, 9.8, 4.0 Hz, 1H), 4.38 (ddd, *J* = 25.3, 9.9, 4.0 Hz, 1H), 4.03–3.96 (m, 1H), 3.76–3.64 (m, 1H), 3.52–3.46 (m, 1H), 3.35–3.27 (m, 1H), 2.06–1.96 (m, 1H), 1.93–1.76 (m, 3H), 1.47 (s, 9H); ^13^C NMR (100 MHz, CDCl_3_) δ 157.9, 85.6 (d, *J* = 170.9 Hz), 80.7, 74.8 (d, *J* = 17.0 Hz), 60.0, 47.4, 28.4, 28.3, 24.2; ^19^F NMR (400 MHz, CDCl_3_) δ −231.3 (td, *J* = 50.0, 23.1 Hz); HRMS (ESI) calculated for [M + Na]^+^ C_11_H_20_FNNaO_3_^+^ was 256.1319 and it was found to be 256.1322; tert-butyl (*S*)-2-((*S*)-2-fluoro-1-hydroxyethyl)pyrrolidine-1-carboxylate (**5a**): [α]^18^_D_ −58.0 (*c* 0.1, CHCl_3_); IR (KBr) ν_max_ 3421, 2981, 2948, 1657, 1405, 1170 cm^−1^; ^1^H NMR (400 MHz, CDCl_3_) δ 4.50–4.41 (m, 1H), 4.38–4.29 (m, 1H), 4.01–3.92 (m, 2H), 3.53–3.45 (m, 1H), 3.30–3.22 (m, 1H), 2.05–1.85 (m, 3H), 1.82–1.74 (m, 1H), 1.46 (s, 9H); ^13^C NMR (100 MHz, CDCl_3_) δ 156.4, 84.5 (d, *J* = 169.1 Hz), 80.5, 71.9 (d, *J* = 18.6 Hz), 60.3, 47.7, 28.5, 27.9, 24.0; ^19^F NMR (400 MHz, CDCl_3_) δ −228.06 (td, *J* = 49.8, 16.2 Hz); HRMS (ESI) calculated for [M + Na]^+^ C_11_H_20_FNNaO_3_^+^ was 256.1319 and it was found to be 256.1322.

*(S)-1-(N-((2R,4S)-2,4-dimethyldec-9-ynoyl)-N-methyl-L-alanyl)-N-((S)-1-(((S)-1-(((S)-1-(((S)-1-((S)-2-((S)-2-((R)-2-fluoro-1-hydroxyethyl)pyrrolidine-1-carbonyl)pyrrolidin-1-yl)-1-oxo-3-phenylpropan-2-yl)(methyl)amino)-3-methyl-1-oxobutan-2-yl)(methyl)amino)-3-methyl-1-oxobutan-2-yl)(methyl)amino)-3-methyl-1-oxobutan-2-yl)-N-methylpyrrolidine-2-carboxamide* (**2b**) 

Compound **2b** was obtained following the procedure for the preparation of compound **2a** we reported previously (65%), colorless oil: [α]^18^_D_ −232.0 (c 0.1, CHCl_3_); IR (KBr) ν_max_ 3446, 2965, 2935, 2114, 1639, 1445, 1098 cm^−1^; ^1^H NMR (400 MHz, CD_3_OD) δ 7.33–7.21 (m, 5H), 5.88 (dd, *J* = 11.6, 4.3 Hz, 1H), 5.30 (q, *J* = 7.0 Hz, 1H), 5.06 (d, *J* = 10.8 Hz, 1H), 5.03 (d, *J* = 10.6 Hz, 1H), 4.93 (d, *J* = 10.7 Hz, 1H), 4.85 (dd, *J* = 8.6, 4.1 Hz, 1H), 4.73 (dd, *J* = 8.6, 4.0 Hz, 1H), 4.53, 4.42 (dd, *J* = 9.9, 3.4 Hz, 1H), 4.34, 4.21 (dd, *J* = 9.9, 6.8 Hz, 1H), 4.31–4.27 (m, 1H), 4.06–3.96 (m, 1H), 3.89–3.83 (m, 1H), 3.79–3.73 (m, 1H), 3.67–3.55 (m, 4H), 3.16–2.94 (m, 3H), 3.10 (s, 3H), 3.03 (s, 3H), 2.96 (s, 3H), 2.93 (s, 3H), 2.35–2.25 (m, 5H), 2.21–2.17 (m, 4H), 2.15 (s, 3H), 2.05–1.92 (m, 8H), 1.75–1.68 (m, 1H), 1.53–1.42 (m, 6H), 1.36–1.32 (m, 2H), 1.28 (d, *J* = 7.0 Hz, 3H), 1.15–1.07 (m, 1H), 0.91 (d, *J* = 7.2 Hz, 3H), 0.89 (d, *J* = 6.6 Hz, 3H), 0.87 (d, *J* = 7.9 Hz, 3H), 0.83 (d, *J* = 6.4 Hz, 3H), 0.76 (d, *J* = 6.7 Hz, 3H), 0.65 (d, *J* = 6.8 Hz, 3H); ^13^C NMR (100 MHz, CD_3_OD) δ 179.3, 174.7, 174.1, 172.1, 171.8, 171.1, 170.9, 170.5, 138.3, 130.5, 129.6, 128.0, 86.4 (d, *J* = 168.4 Hz), 85.0, 74.1 (d, *J* = 18.4 Hz), 69.5, 60.1, 60.0, 59.8, 58.8, 56.6, 52.5, 48.8, 48.6, 48.4, 42.5, 37.5, 35.1, 34.8, 31.8, 31.4, 31.3, 30.9, 30.8, 30.2, 29.9, 29.8, 28.2, 28.1, 28.07, 27.0, 25.82, 25.76, 25.6, 20.4, 20.2, 20.1, 19.9, 18.9, 18.8, 18.3, 18.0, 17.8, 14.2; ^19^F NMR (400 MHz, CDCl_3_) δ −232.28 (td, *J* = 51.1, 20.8 Hz); HRMS (ESI) calculated for [M + Na]^+^ C_60_H_95_FN_8_NaO_9_^+^ was 1113.7098 and it was found to be 1113.7102.

*(S)-1-(N-((2R,4S)-2,4-dimethyldec-9-ynoyl)-N-methyl-L-alanyl)-N-((S)-1-(((S)-1-(((S)-1-(((S)-1-((S)-2-((S)-2-(2-fluoroacetyl)pyrrolidine-1-carbonyl)pyrrolidin-1-yl)-1-oxo-3-phenylpropan-2-yl)(methyl)amino)-3-methyl-1-oxobutan-2-yl)(methyl)amino)-3-methyl-1-oxobutan-2-yl)(methyl)amino)-3-methyl-1-oxobutan-2-yl)-N-methylpyrrolidine-2-carboxamide* (**1b**)

Compound **1b** was obtained following the procedure for the preparation of compound **1a** we reported previously (75%), white solid: [α]^18^_D_ −216.6 (c 0.1, CHCl_3_); IR (KBr) ν_max_ 2965, 2935, 2114, 1741, 1639, 1444, 1098 cm^−1^; ^1^H NMR (400 MHz, CD_3_OD) δ 7.32–7.22 (m, 5H), 5.88 (dd, *J* = 11.6, 4.4 Hz, 1H), 5.30 (q, *J* = 7.0 Hz, 1H), 5.18 (d, *J* = 2.2 Hz, 1H), 5.06 (d, *J* = 2.2 Hz, 1H), 5.06 (d, *J* = 10.8 Hz, 1H), 5.02 (d, *J* = 10.6 Hz, 1H), 4.93 (d, *J* = 10.7 Hz, 1H), 4.84 (dd, *J* = 8.6, 4.0 Hz, 1H), 4.78–4.74 (m, 1H), 4.72 (dd, *J* = 8.7, 3.9 Hz, 1H), 3.89–3.81 (m, 1H), 3.77–3.70 (m, 1H), 3.67–3.54 (m, 4H), 3.14–2.94 (m, 3H), 3.10 (s, 3H), 3.03 (s, 3H), 2.96 (s, 3H), 2.93 (s, 3H), 2.33–2.24 (m, 5H), 2.21–2.16 (m, 4H), 2.14 (s, 3H), 2.09–1.94 (m, 8H), 1.75–1.68 (m, 1H), 1.53–1.41 (m, 6H), 1.37–1.31 (m, 2H), 1.27 (d, *J* = 7.0 Hz, 3H), 1.17–1.11 (m, 1H), 1.07 (d, *J* = 6.7 Hz, 3H), 0.91 (d, *J* = 7.2 Hz, 3H), 0.89 (d, *J* = 6.5 Hz, 3H), 0.88 (d, *J* = 6.4 Hz, 3H), 0.83 (d, *J* = 6.4 Hz, 6H), 0.76 (d, *J* = 6.7 Hz, 3H), 0.65 (d, *J* = 6.8 Hz, 3H); ^13^C NMR (100 MHz, CD_3_OD) δ 205.4 (d, *J* = 16.8 Hz), 179.3, 174.7, 172.5, 172.1, 171.8, 171.1, 170.8, 170.5, 138.3, 130.5, 129.6, 128.1, 86.3(d, *J* = 180.4 Hz), 85.0, 69.6, 62.7, 60.0, 59.8, 58.8, 56.6, 52.5, 48.9, 48.6, 48.3, 42.5, 37.5, 35.1, 34.8, 31.8, 31.4, 31.3, 30.9, 30.8, 30.2, 29.8, 29.3, 28.7, 28.2, 28.0, 27.0, 26.0, 25.8, 20.4, 20.2, 20.1, 19.9, 18.9, 18.8, 18.3, 18.0, 17.8, 14.2; ^19^F NMR (400 MHz, CDCl_3_) δ −233.7 (t, *J* = 50.0 Hz); HRMS (ESI) calculated for [M + Na]^+^ C_60_H_93_FN_8_NaO_9_^+^ was 1111.6942 and it was found to be 1111.6946.

### 3.3. Cell Culture

Human leukemia cell line HL60 was purchased from the American Type Culture Collection (USA). Human lung cancer cell lines H820, H1299, and A549 were purchased from the National Infrastructure of Cell Line Resource (Beijing, China). The H1688 cell line was donated by Tianjin Medical University Cancer Institute and Hospital. These cancer cells were cultured in 1640 medium supplemented with 10% FBS and 100 units of penicillin-streptomycin under 5% CO_2_ at 37 °C.

### 3.4. CCK-8 Assay

Cells were seeded with a density of 3000–10,000 cells/100 μL/well into a 96-well plate. After 18–24 h, the compounds were diluted to different concentrations and added to the 96-well plate. After incubation for 72 h, the CCK-8 reagent was added and incubated at 37 °C for an additional 1–4 h. The absorbance was measured at 450 nm using a microplate reader. The 50% inhibitory concentration (IC_50_) values were calculated using GraphPad Prism 5 (GraphPad, San Diego, CA, USA).

### 3.5. Cell Cycle Assay

The H820 cells were seeded into a 6-well plate with a density of 5 × 10^5^ cells/3 mL/well. Compounds **2b** and **1a** at different concentrations (0 μM, 4 μM, 8 μM, 12 μM, and 16 μM) were added and incubated for 48 h. After being collected, the cells were immobilized with 75% ethanol on ice for 2 h. The cells were washed twice with PBS and incubated with propidium iodide (PI; Sigma, St. Louis, MO, USA) for 15 min. The cells were analyzed by flow cytometry (NovoCyte, ACEA, San Diego, CA, USA).

### 3.6. Apoptosis Assay

The H820 cells were seeded into a 24-well plate with a density of 1 × 10^5^ cells/1 mL/well. Compounds **2b** and **1a** at different concentrations (0 μM, 4 μM, 8 μM, 12 μM, and 16 μM) were added and incubated for 48 h. After being collected, the cells were incubated with Annexin-V-FITC and 7-aminoactinomycin D (7AAD) in the dark for 15 min. The cells were analyzed by flow cytometry (NovoCyte, ACEA, San Diego, CA, USA).

## 4. Conclusions

By coupling and DMP oxidation, we synthesized jahanyne and its fluoro analogs. Preliminary bioassays showed that **2b** showed increased activities towards H1688 cells, H1299 cells, and H820 cells compared to jahanyne. However, **1b** (FMK-jahanyne) exhibited decreased activities to varying degrees against most of the cells tested. The results indicated that the unique terminal OEP moiety might not interact with its target protein in a covalent manner. Jahanyne and compound **2b** behaved in a similar manner in regulating the G0/G1 phase cell cycle. With an alkynyl group, jahanyne itself could act as a probe to investigate its detailed mode of action. The target verification, together with its medicinal work, is ongoing and will be reported in due course.

## Figures and Tables

**Table 1:** Anticancer activities of compounds against human cancer cells.

Compounds	IC_50_ (μM) ^a^				
	HL60	H1688	H1299	H820	A549
**2a**	19.53 ± 4.73	17.73 ± 2.80	16.73 ± 3.03	9.01 ± 1.75	16.68 ± 1.05
**2b**	29.68 ± 9.41	13.47 ± 3.10	10.98 ± 1.31	7.64 ± 1.70	14.65 ± 2.06
**1a** (jahanyne)	13.98 ± 1.84	21.90 ± 3.26	18.70 ± 1.55	11.48 ± 1.87	17.11 ± 2.79
**1b**	14.70 ± 0.56	29.35 ± 4.37	28.56 ± 9.15	19.38 ± 1.87	13.83 ± 2.21

^a^ The IC_50_ is the concentration of compound required to achieve 50% inhibition of tumor cells. All values are presented as the mean ± SD of three independent experiments.

## Supplementary Materials

The following are available online at https://www.mdpi.com/1660-3397/18/3/176/s1, Figures S1–S19: NMR, COSY and HSQC spectrum.
