# Supplementary material for: Design, Synthesis and Biological Evaluation of Jahanyne Analogs as Cell Cycle Arrest Inducers
Source: Mar Drugs. 2020 Mar 23;18(3):176. doi: 10.3390/md18030176 (PMC7142928; doi:10.3390/md18030176)
Supplement: Supplementary file 1 [file marinedrugs-18-00176-s001.pdf]

Design, Synthesis and Biological Evaluation of Jahanyne Analogues as Cell Cycle Arrest Inducers

Baijun Ye [1], Jianmiao Gong [2], Qiuying Li [2], Shiqi Bao [2], Xuemei Zhang [2], Jing Chen [2], Qing Meng [1], Bolin Chen[1], Peng Jiang [1], Liang Wang [1,*] and Yue Chen[1, 3, *]

[1] The State Key Laboratory of Medicinal Chemical Biology, College of Pharmacy and Tianjin Key Laboratory of Molecular Drug Research, Nankai University, Tianjin 300350, PR China; yebaijunts@126.com (B.Y.); mengqing19920220@163.com (Q.M.); 2120150647@mail.nankai.edu.cn (B.C.); jiang1921372889@126.com (P.J.);

[2] Accendatech Co., Ltd., Tianjin 300384, PR China; jianmiao.gong@accendatech.com (J.G.); liqiuying@mail.nankai.edu.cn (Q.L.); shiqi.bao@accendatech.com (S.B.); xuemei.zhang@accendatech.com (X.Z.); chenjing@accendatech.com (J.C.);

[3] Collaborative Innovation Center of Chemical Science and Engineering, Tianjin 300350, PR China;

* Correspondence: lwang@nankai.edu.cn (L.W.); yuechen@nankai.edu.cn (Y.C.); Tel.: +86-22-85358387

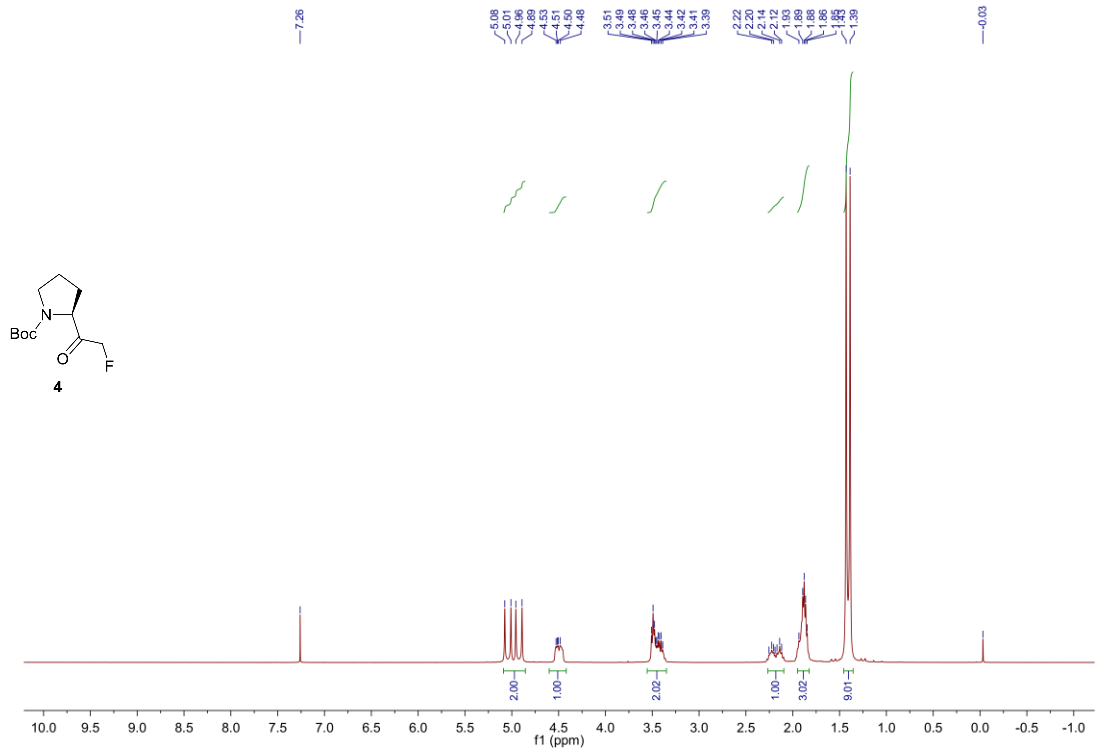

Figure S1. ^{1}H NMR (400 MHz, $CDCl_3$) spectrum of compound **4**.

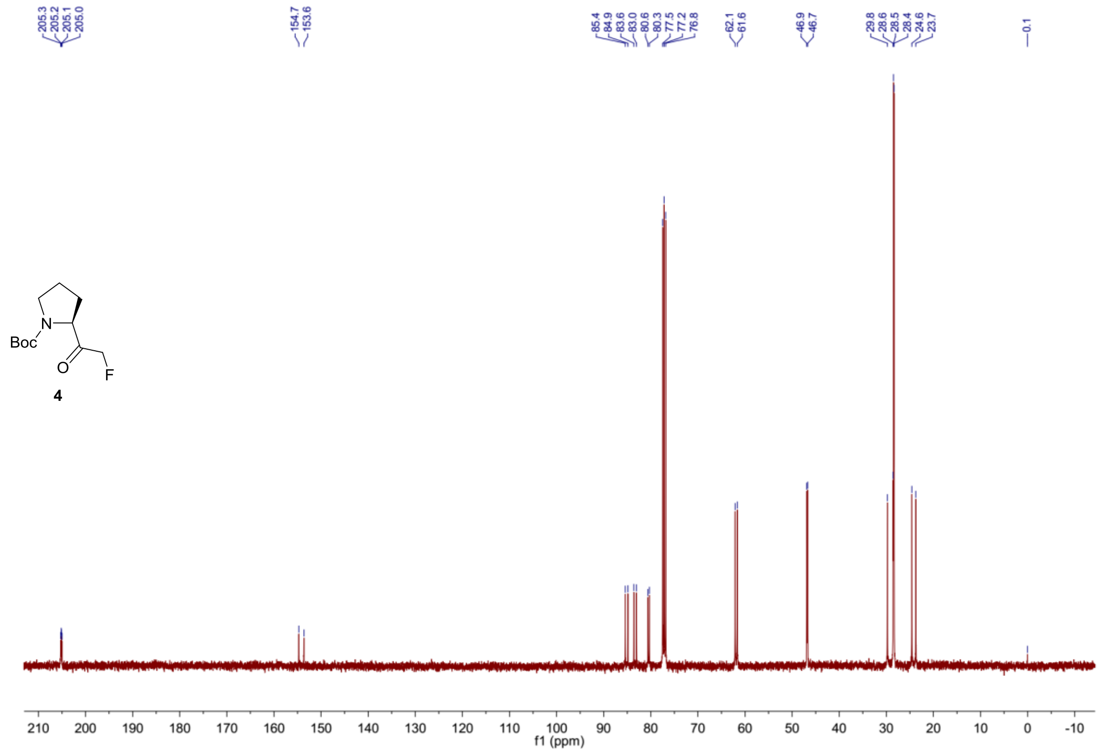

Figure S2. ^{13}C NMR (100 MHz, $CDCl_3$) spectrum of compound **4**.

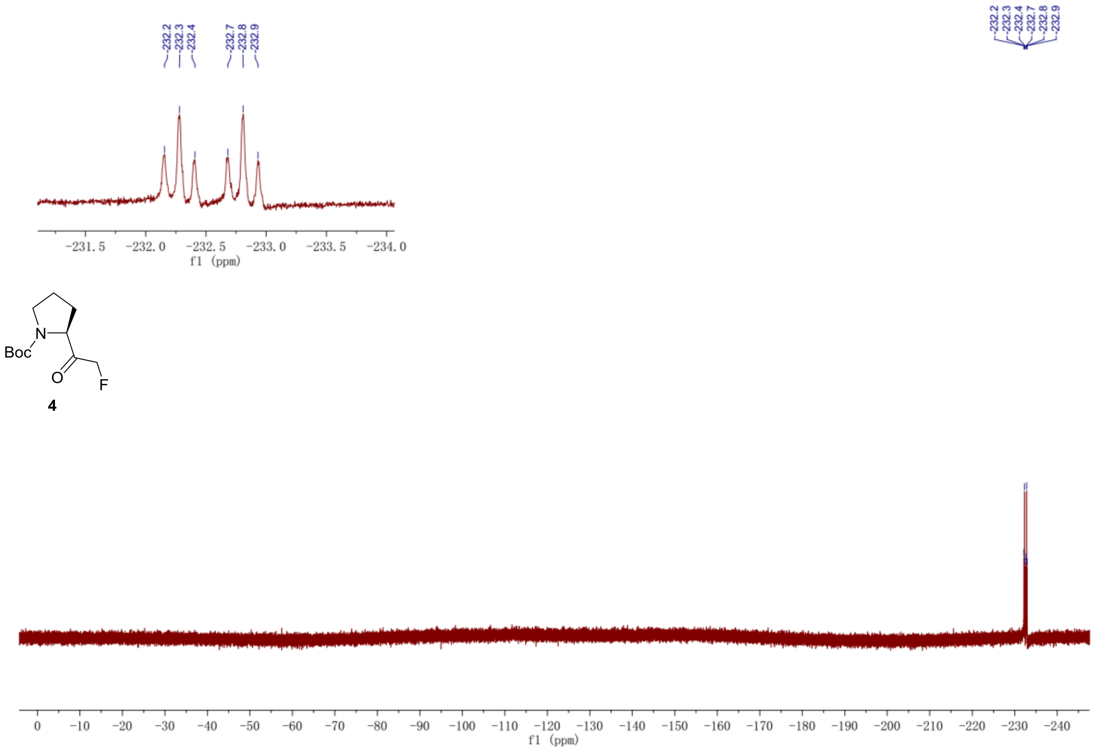

Figure S3. ^{19}F NMR (400 MHz, $CDCl_3$) spectrum of compound **4**.

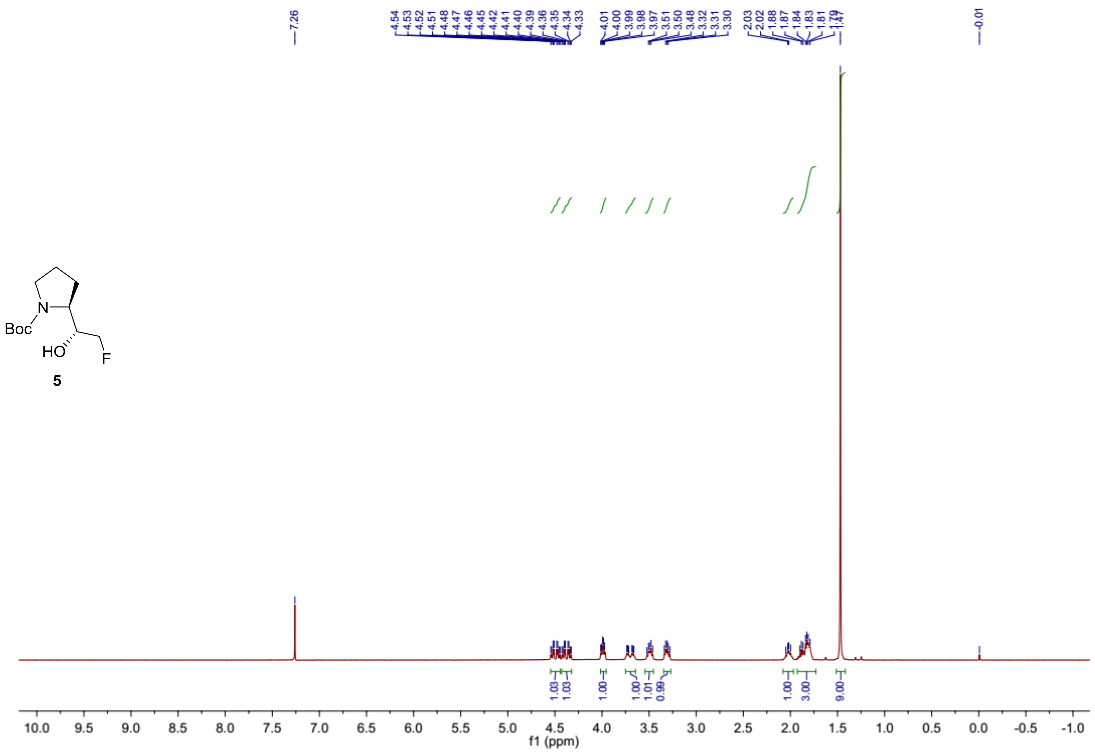

Figure S4. ^{1}H NMR (400 MHz, $CDCl_3$) spectrum of compound **5**.

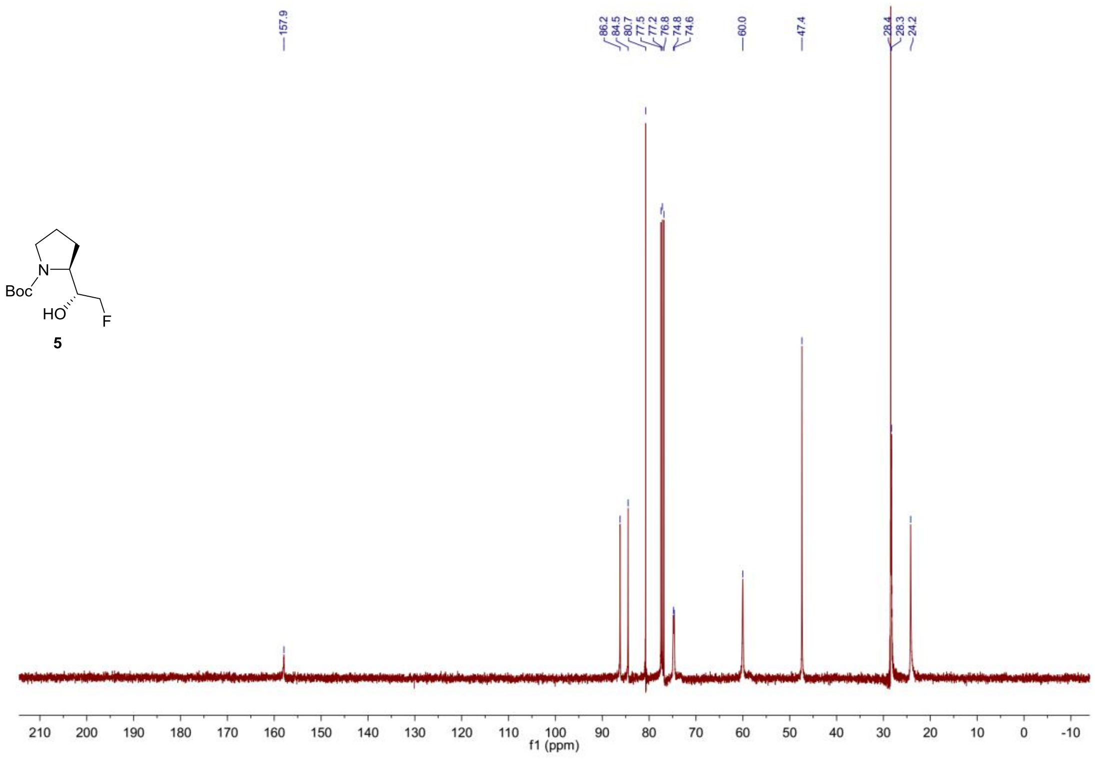

Figure S5. ^{13}C NMR (100 MHz, $CDCl_3$) spectrum of compound **5**.

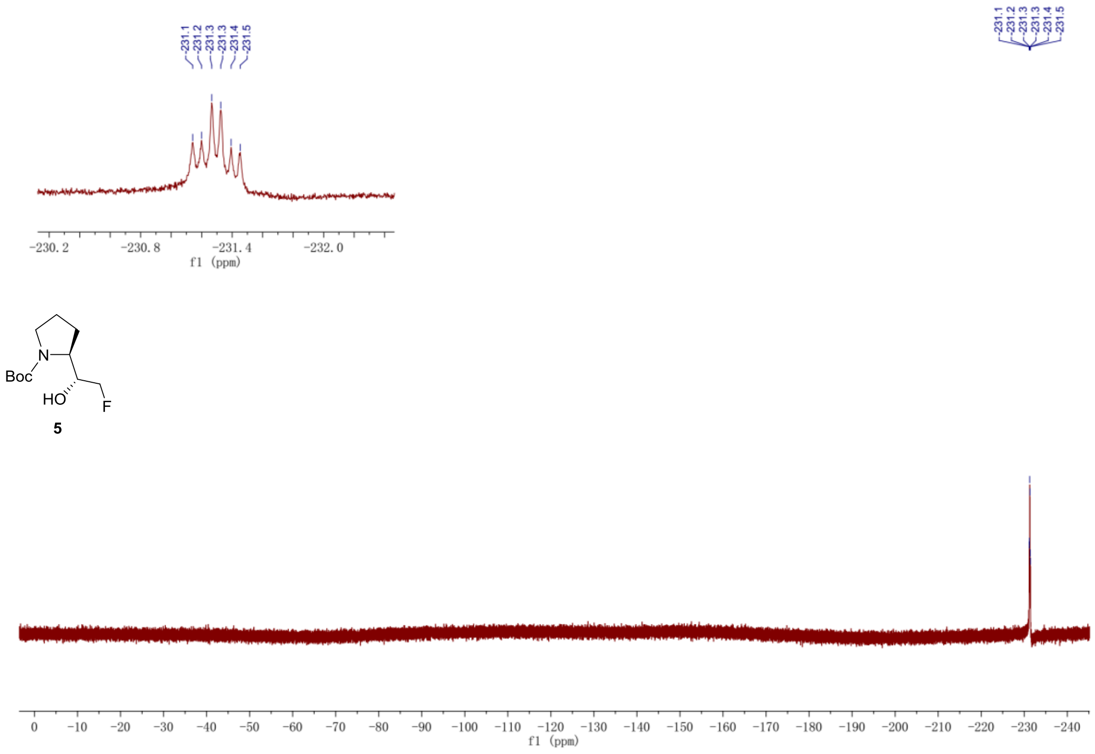

Figure S6. ^{19}F NMR (400 MHz, $CDCl_3$) spectrum of compound **5**.

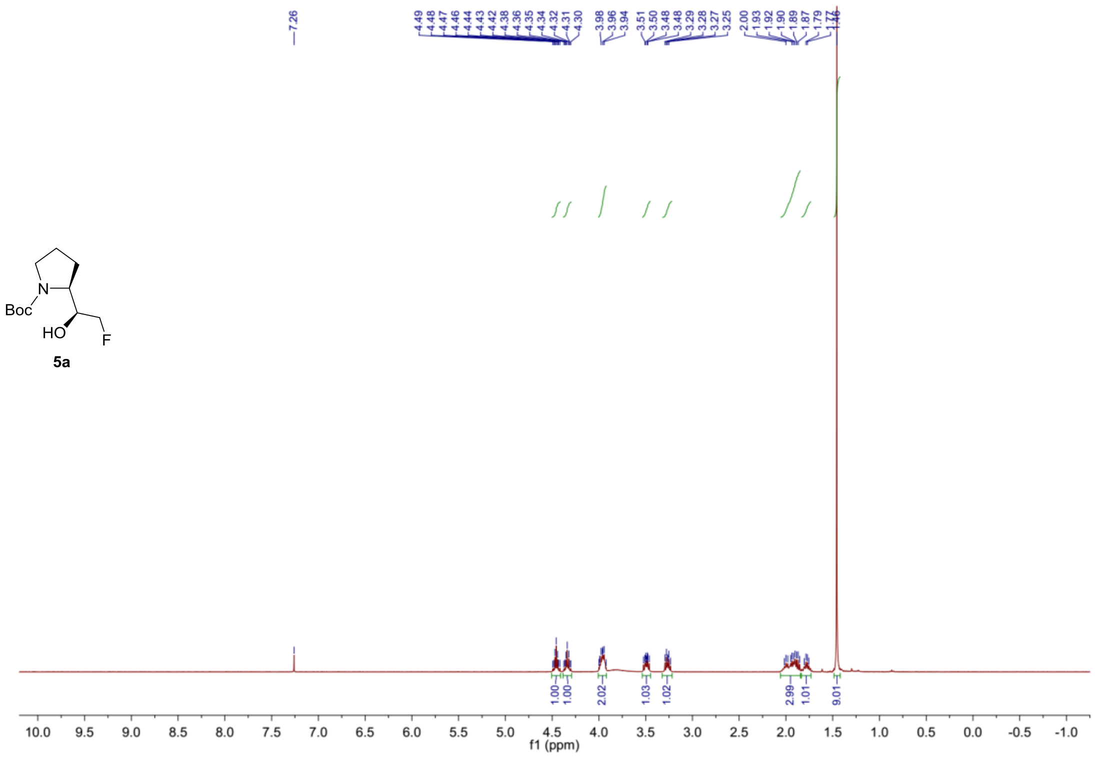

Figure S7. ^{1}H NMR (400 MHz, $CDCl_3$) spectrum of compound **5a**.

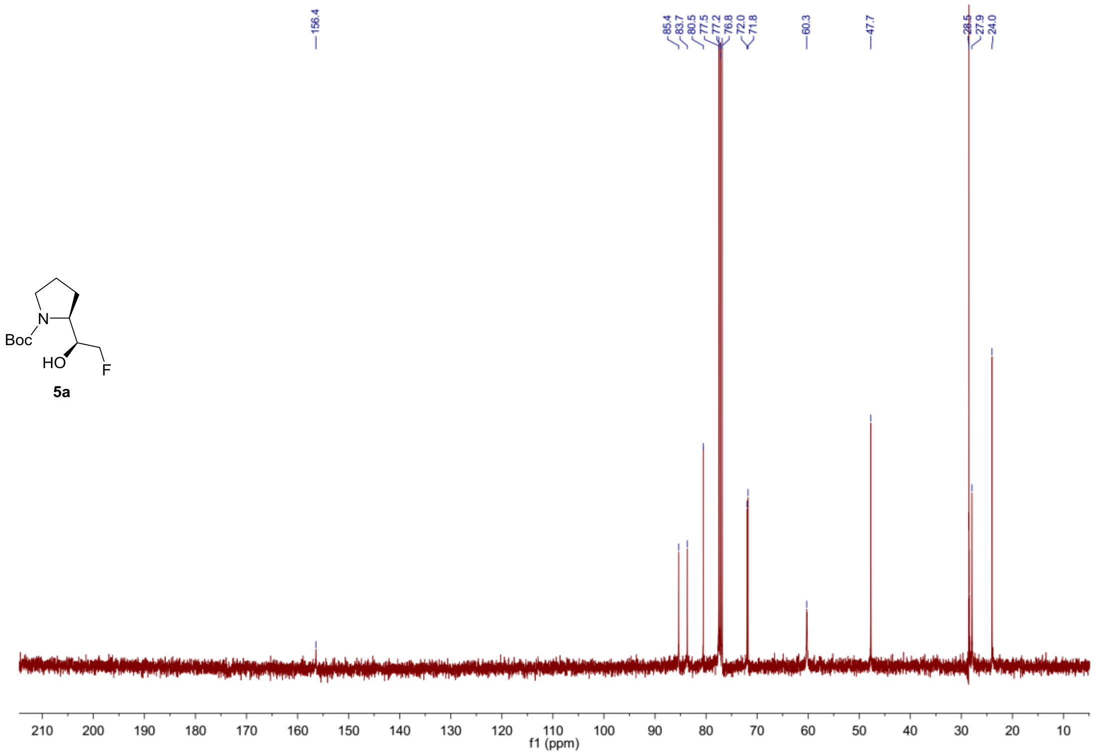

Figure S8. ^{13}C NMR (100 MHz, $CDCl_3$) spectrum of compound **5a**.

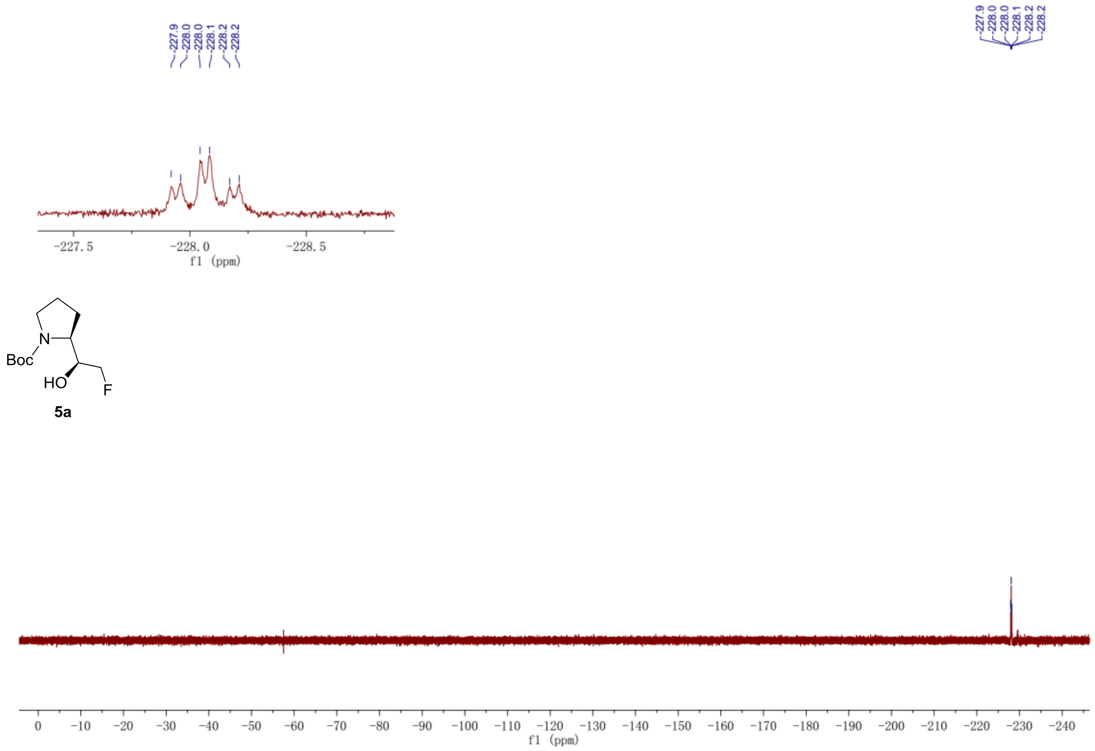

Figure S9. ^{19}F NMR (400 MHz, $CDCl_3$) spectrum of compound **5a**.

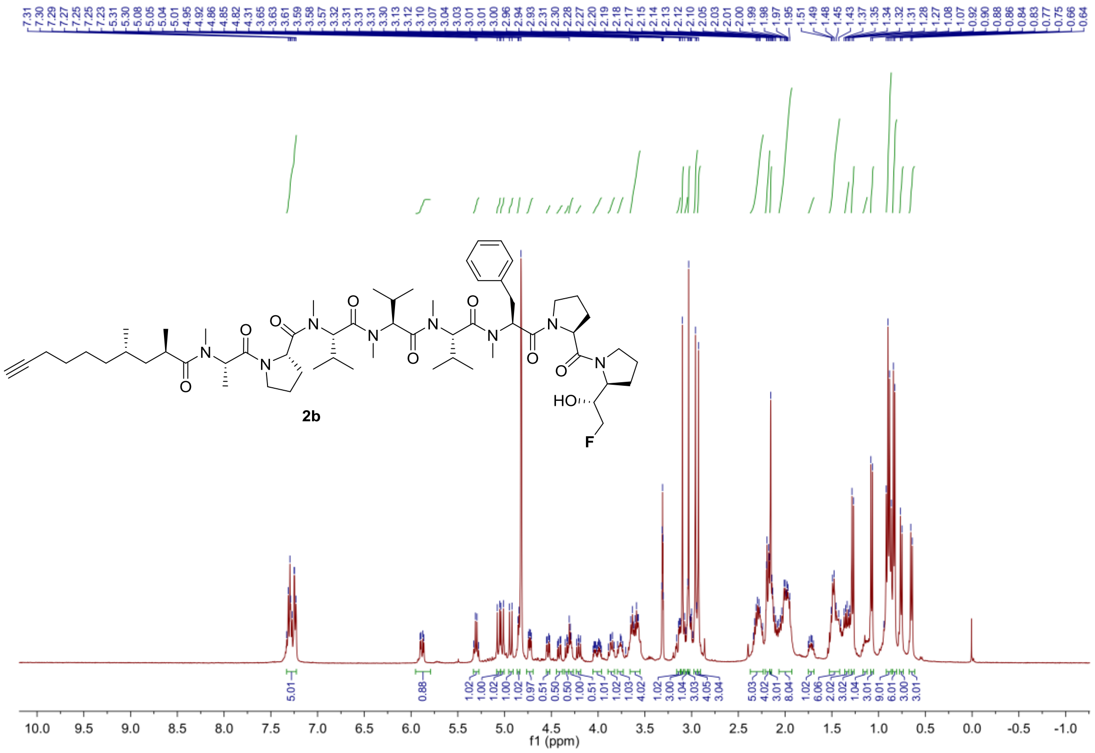

Figure S10. ^{1}H NMR (400 MHz, CD_3OD) spectrum of compound **2b**.

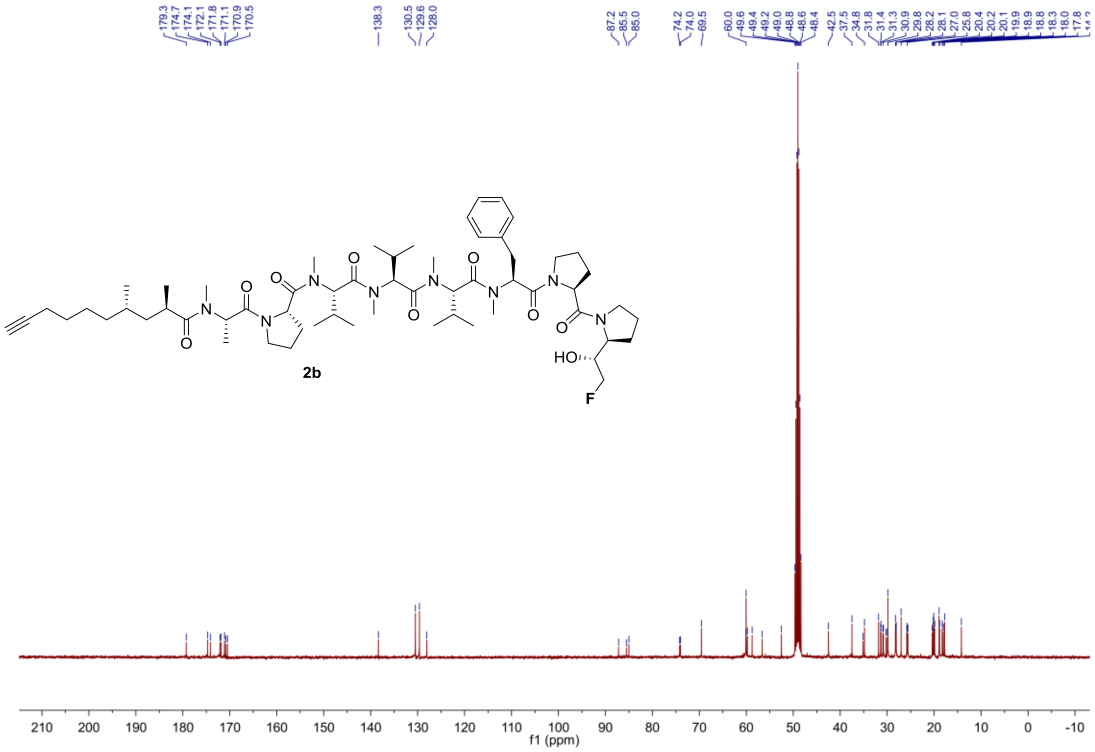

Figure S11. ^{13}C NMR (100 MHz, CD_3OD) spectrum of compound **2b**.

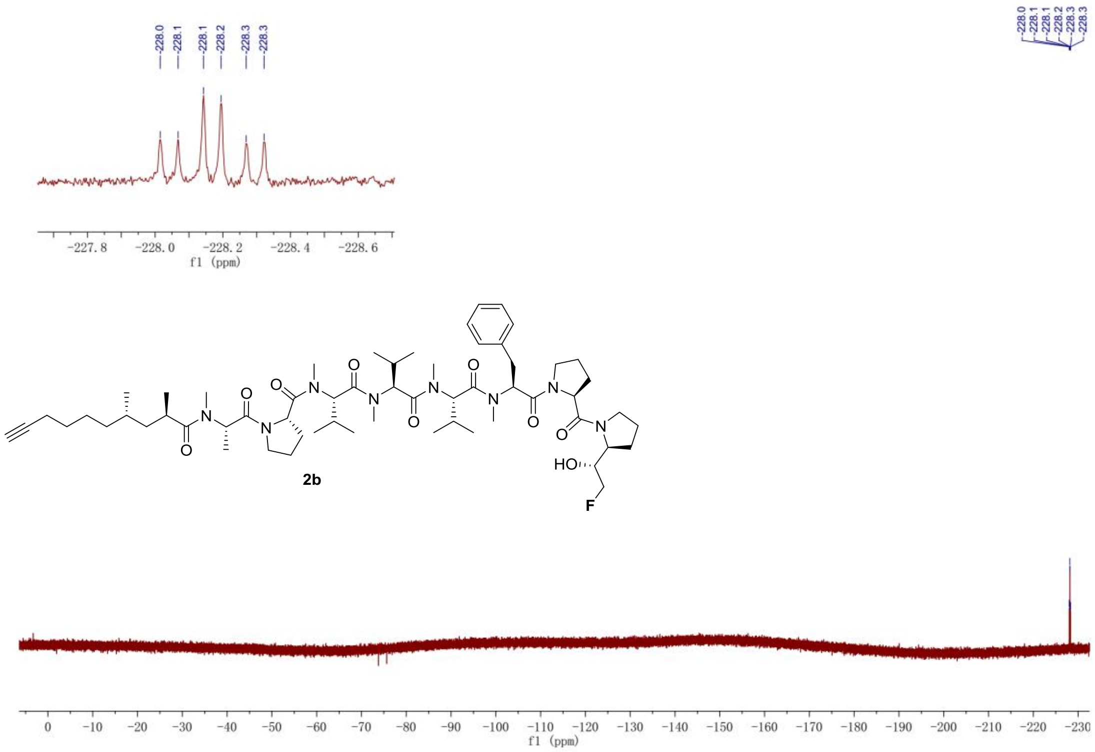

Figure S12. ^{19}F NMR (400 MHz, CD_3OD) spectrum of compound **2b**.

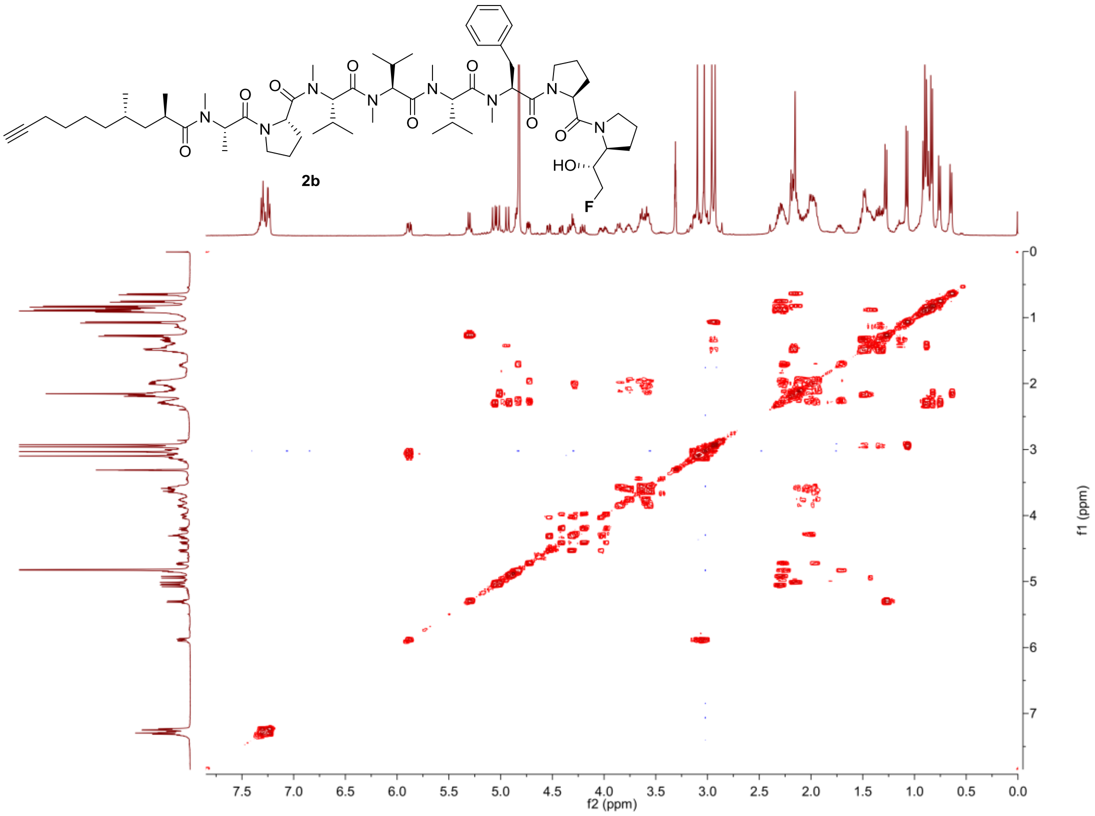

Figure S13. COSY (^{1}H, 400 MHz, CD_3OD spectrum of compound **2b**.

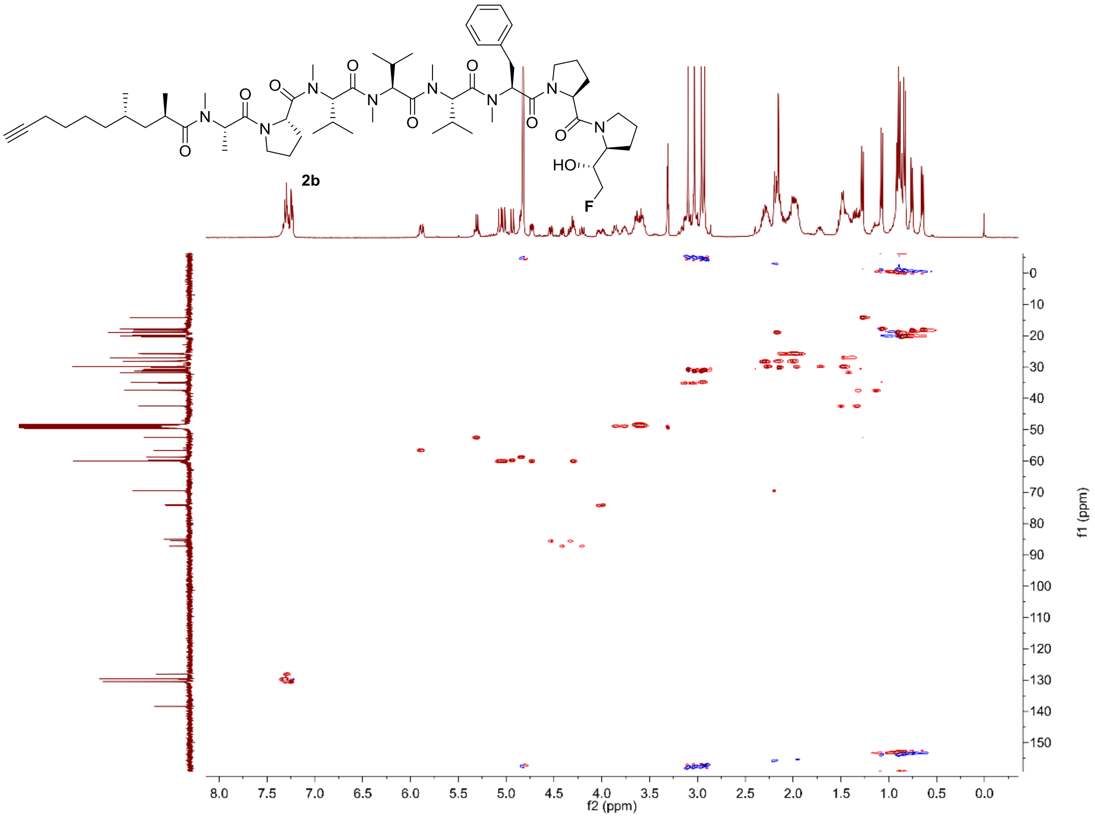

Figure S14. HSQC (^{1}H, 400 MHz, ^{13}C, 100 MHz, CD_3OD) spectrum of compound **2b**.

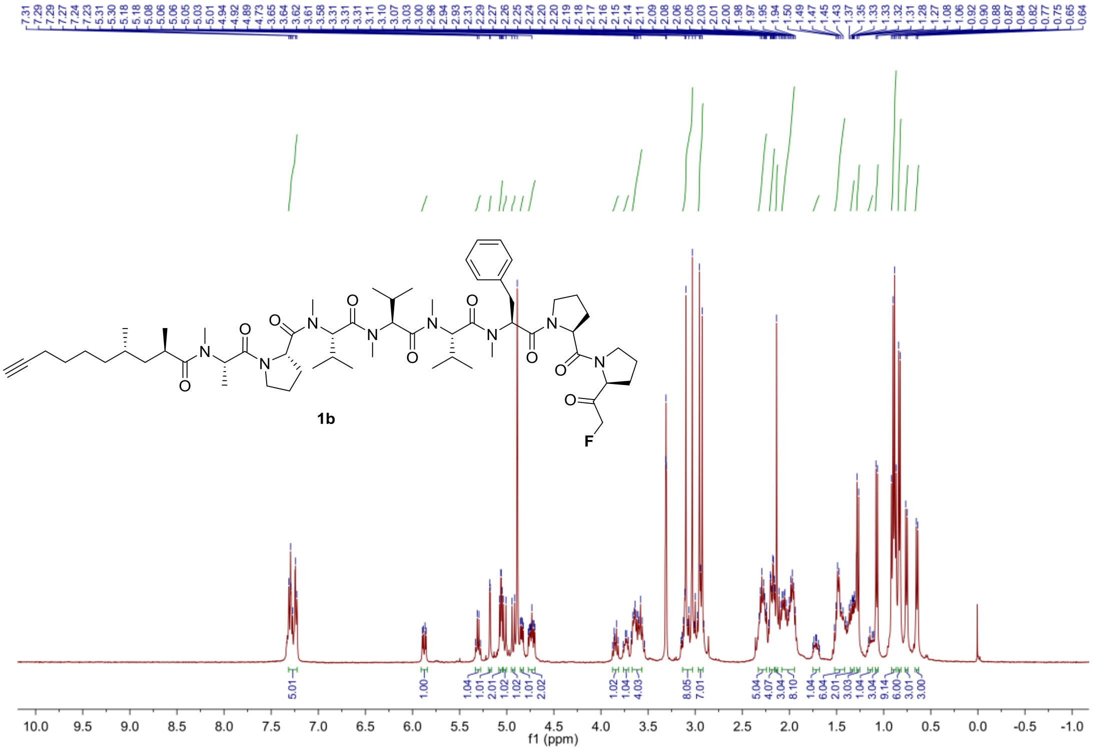

Figure S15. ^{1}H NMR (400 MHz, CD_3OD) spectrum of compound **1b**

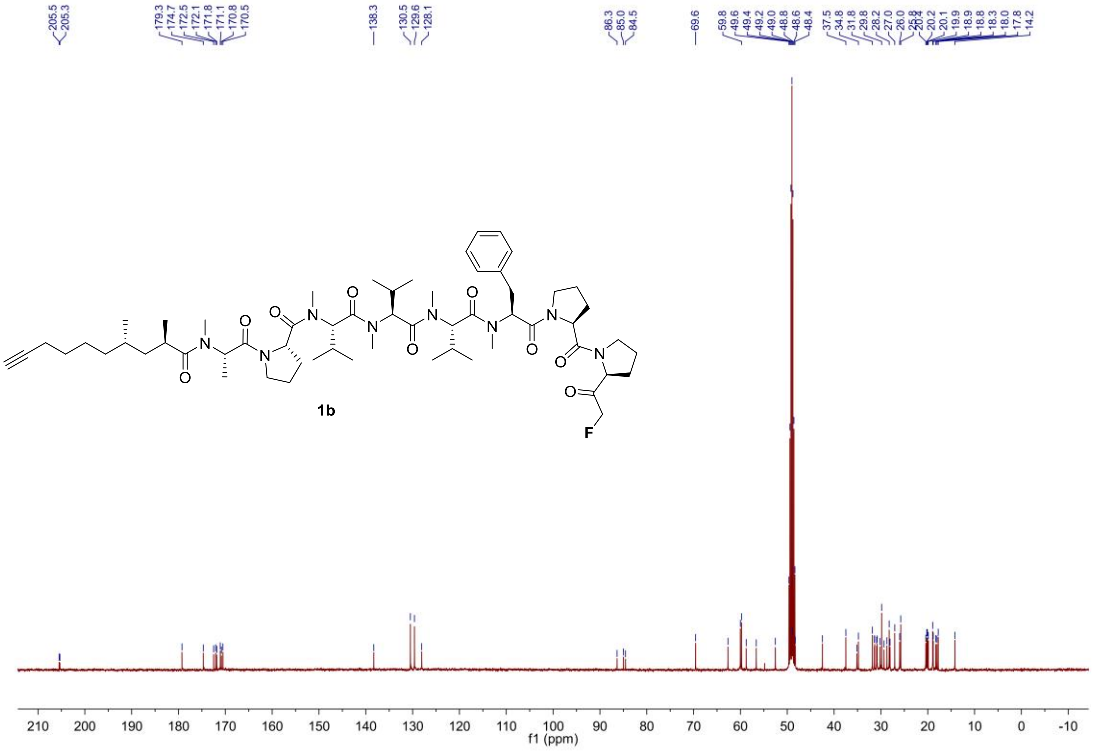

Figure S16. ^{13}C NMR (100 MHz, CD_3OD) spectrum of compound **1b**.

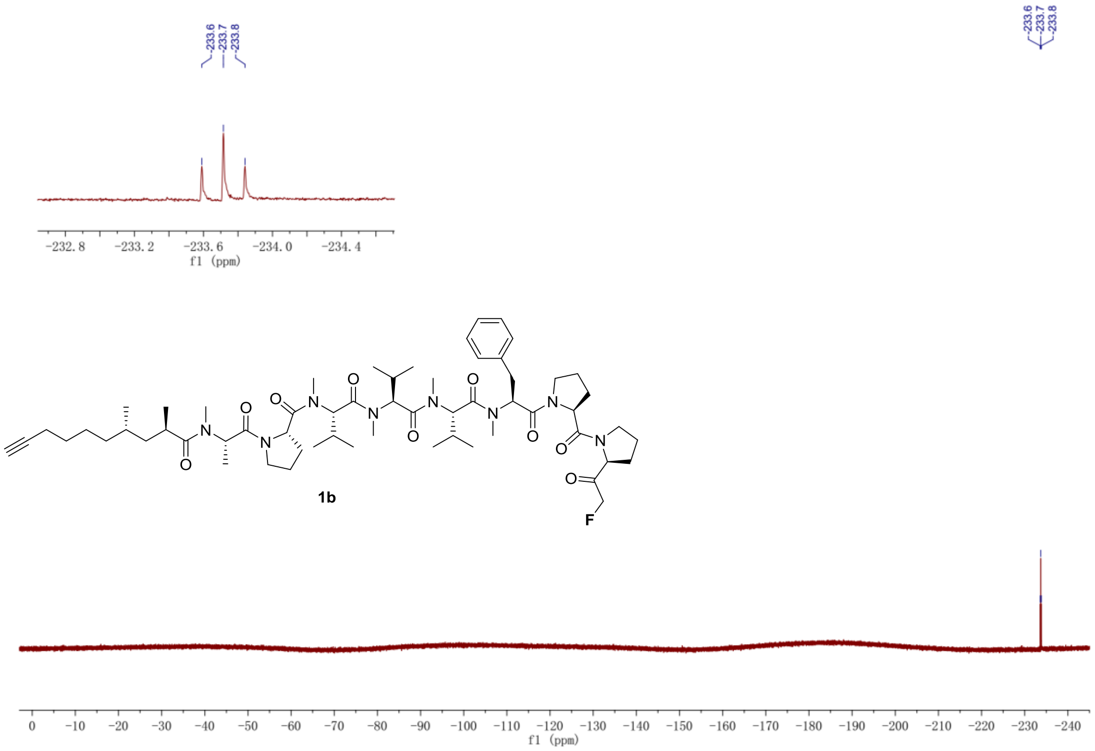

Figure S17. ^{19}F NMR (400 MHz, CD_3OD) spectrum of compound **1b**.

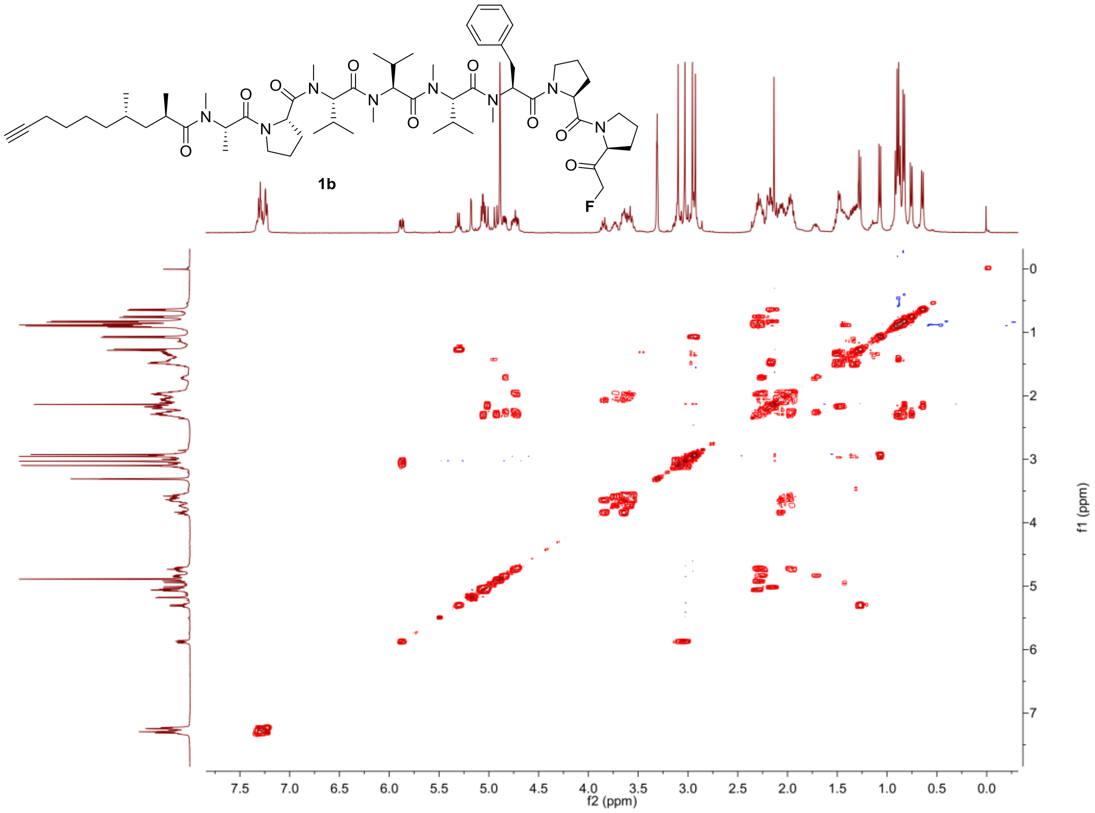

Figure S18. COSY (^{1}H, 400 MHz, CD_3OD) spectrum of compound **1b**.

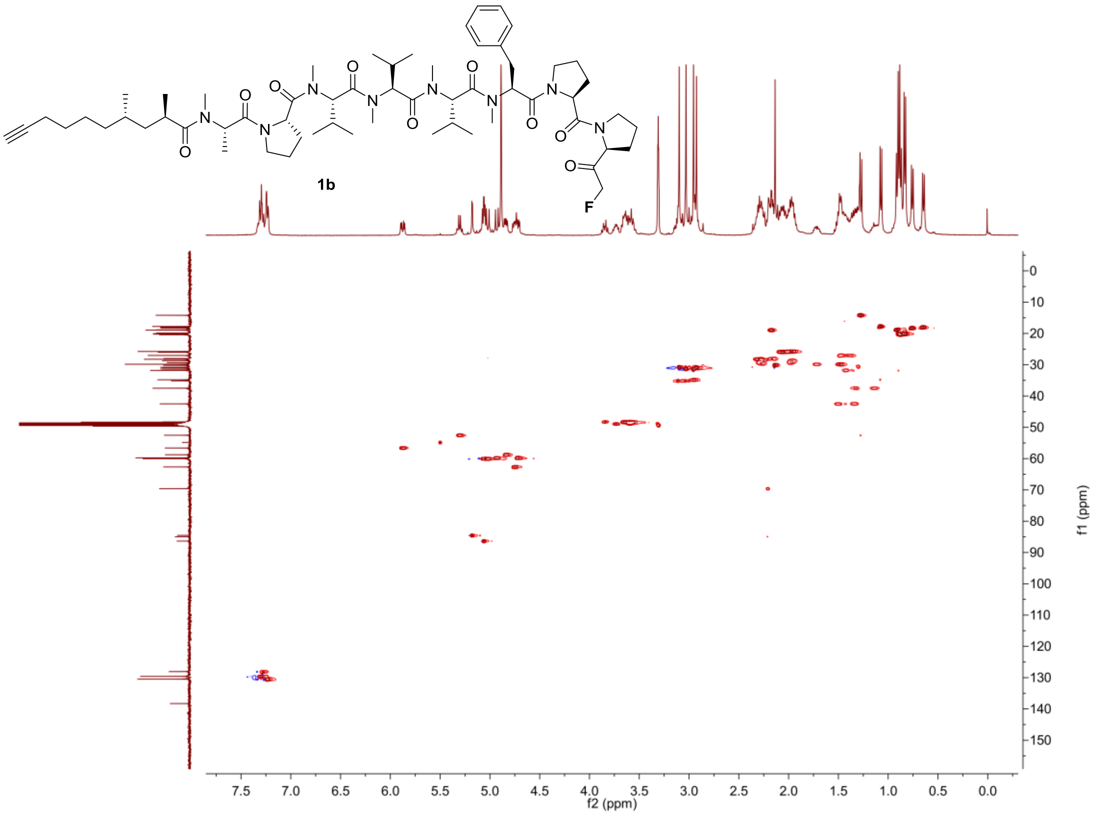

Figure S19. HSQC (^{1}H, 400 MHz, ^{13}C, 100 MHz, CD_3OD) spectrum of compound **1b**.
